# Correction to: Requeening queenright honey bee colonies with queen cells in honey supers

**DOI:** 10.1093/jisesa/iead103

**Published:** 2024-02-09

This is a correction to: Leslie A Holmes, Jeffery D Kearns, Lynae P Ovinge, Patricia Wolf Veiga, Shelley E Hoover, Requeening queenright honey bee colonies with queen cells in honey supers, *Journal of Insect Science*, Volume 23, Issue 5, September 2023, 20, https://doi.org/10.1093/jisesa/iead091

In the original publication of this manuscript, a section was erroneously inserted for **Supplementary Data** in the online version. In the absence of such data, this section is now removed. There was a link in the caption to Figure 1: this should read: “(Table 1)” and hyperlink to the said table in the article, instead of reading: “(Supplementary Table 1)” and linking through to a repeated image of Figure 1.

These errors have been corrected in the article.

